# Guanxinning Injection Combined With Ischemic Postconditioning Attenuate Myocardial Ischemic Reperfusion Injury in Chronic Renal Failure Rats by Modulating Mitochondrial Dynamics

**DOI:** 10.3389/fcvm.2022.905254

**Published:** 2022-05-30

**Authors:** Anzhu Wang, Dawu Zhang, Jiangang Liu, Huijing Yan, Pei Zhang, Hui Yuan, Xiaochang Ma

**Affiliations:** ^1^Xiyuan Hospital, China Academy of Chinese Medical Sciences, Beijing, China; ^2^Graduate School, China Academy of Chinese Medical Sciences, Beijing, China; ^3^National Clinical Research Center for Chinese Medicine Cardiology, Beijing, China

**Keywords:** ischemia-reperfusion injury, chronic renal failure, extracts of *Salvia miltiorrhiza Bge.* (Danshen) and *Ligusticum chuanxiong Hort.* (Chuanxiong), Guanxinning injection, mitochondrial dynamics

## Abstract

**Purpose:**

*Salvia miltiorrhiza Bge.* (Danshen, DS) and *Ligusticum chuanxiong Hort.* (Chuanxiong, CX) have been widely used in traditional Chinese medicine to prevent and treat myocardial ischemia and renal insufficiency, and their extracts (Guanxinning injection, GXN) have been reported to exhibit antioxidant, anti-inflammatory, and anti-ischemia-reperfusion injury properties. It is well-established that ischemic postconditioning (IPOC) can protect against myocardial ischemia-reperfusion (I/R) injury in rats with chronic renal failure (CRF). However, little is known on whether GXN combined with IPOC may affect myocardial I/R injury in CRF rats. We sought to observe the effect of GXN combined with IPOC on myocardial I/R injury in CRF rats by quantifying changes in the expression of proteins related to mitochondrial dynamics.

**Materials and Methods:**

In a survey, 90 Wistar rats were randomly divided into 6 groups (15 rats per group): CRF group, I/R group, comorbid group (CRF + I/R), IPOC group, IPOC + GXN group and the sham group. Changes in blood myocardial injury markers, urea, and creatinine were analyzed. Heart tissues were harvested for histomorphometry and western blotting when rats were sacrificed. Myocardial infarction area was measured by Evans blue and Triphenyltetrazolium chloride solution staining. The expressions of mitochondrial fission relative proteins (DRP1 and FIS1) and mitochondrial fusion relative proteins (OPA1 and MFN1) were detected by western blotting.

**Results:**

IPOC could significantly decrease myocardial injury markers and myocardial area of necrosis (AN)/area at risk (AAR) of the comorbid model rats. Further results showed that GXN combined with IPOC could significantly reduce CK-MB levels and myocardial AN/AAR in comorbid model rats compared with the IPOC group. Meanwhile, both IPOC and IPOC + GXN significantly reduced DRP1 levels and increased the MFN1 and OPA1 protein levels in the comorbid model rats. However, compared with the IPOC group, MFN1 and OPA1 protein levels increased significantly in the IPOC + GXN group.

**Conclusion:**

Extracts of DS and CX combined with IPOC exert a protective effect against myocardial I/R injury in rats with CRF, mediated by increased expression of mitochondrial fusion proteins (MFN1 and OPA1).

## Introduction

The global burden of chronic kidney disease (CKD) has increased in recent years, with approximately 10% of adults worldwide suffering from some form of CKD, leading to 1.2 million deaths and 28 million lives lost each year. Consistently, the prevalence of chronic renal failure (CRF) has also escalated (1). Despite substantial breakthroughs in revascularization and medicinal treatment, acute myocardial infarction (AMI), and subsequent progression to heart failure (HF) are associated with an increased risk of death owing to ischemia-reperfusion (I/R) injury (2). It is widely acknowledged that cardiovascular diseases such as AMI and CKD exacerbate each other, accounting for the high mortality rates (3). A study demonstrated that compared to patients with normal renal function, cardiovascular mortality was twice as high in patients with stage 3 CKD and three times higher in stage 4 disease (4). Besides, CKD patients reportedly account for 16.5% of AMI survivors (5); current evidence suggests that CKD is linked to poor long-term outcomes in patients with AMI, even after revascularization intervention based on standardized western medicine (6). These findings emphasize the need to explore novel approaches to improve the prognosis of AMI patients with CKD and reduce death or disability rates.

Ischemic postconditioning (IPOC) was first proposed in 2003 by Zhao et al., who found that repeated transient ischemia 60 min before ligation of the descending coronary artery could significantly reduce reperfusion injury in dogs before restoration of continuous coronary blood perfusion (7). In addition to animal studies, IPOC has been shown to reduce myocardial injury in patients following percutaneous coronary intervention (PCI) or coronary artery bypass grafting in clinical trials (8, 9). An increasing body of evidence suggests that IPOC can protect against myocardial I/R injury, reduce myocardial infarction area and improve cardiac function in chronic renal failure (CRF) rats (10). In a previous study, we established that IPOC could reduce the myocardial infarct area caused by I/R and protect cardiac function. Interestingly, the application of *Huoxue* Chinese medicine potentiated the protective effect of IPOC against myocardial injury caused by I/R (11). Moreover, a clinical trial study showed that *Huoxue* Chinese medicine could reduce the primary endpoint events (death, non-fatal myocardial infarction, ischemia-driven revascularization) and secondary endpoint events (readmission for acute coronary syndrome, stroke, HF) for patients with acute coronary syndrome after PCI (12). The subgroup analysis substantiated that standardized treatment with *Huoxue* Chinese medicine combined with western medicine could improve cardiovascular prognosis and renal function of patients with acute coronary syndrome complicated with renal insufficiency after PCI (13). Accordingly, further studies are warranted to assess whether *Huoxue* Chinese medicine combined with IPOC can alleviate myocardial I/R injury in the presence of comorbidities such as CKD.

*Salvia miltiorrhiza Bge.* (Danshen, DS) and *Ligusticum chuanxiong Hort.* (Chuanxiong, CX) are commonly *Huoxue* Chinese medicine during clinical practice. The extracts of DS and CX, which are well-established to protect against myocardial I/R injury, were selected as the research drugs (14). The extracts of DS and CX are compound traditional Chinese medicine (TCM) injections with many functions, including dilating coronary arteries, promoting coronary collateral circulation, accelerating ischemic myocardial repair, protecting ischemic hypoxic myocardium, and treating angina pectoris. It is approved by China’s State Food and Drug Administration (SFDA) for clinical use in patients with coronary artery disease under the trade name Guanxinning (GXN).

Mitochondria are key organelles that participate in the pathophysiology of CKD complicated with AMI (15). Mitochondria are well-recognized to maintain a state of kinetic balance in physiological states, namely continuous division, and fusion. However, during cell injury such as I/R injury, this balance is dysregulated, leading to cell apoptosis or necrosis (16). Indeed, to protect cardiac myocytes, the equilibrium between mitochondrial division and fusion must be maintained. Current evidence suggests that dynamin-related protein-1 (DRP1) and mitochondrial fission protein 1 (FIS1), an adaptor protein anchored in the outer membrane, are involved in mitochondrial division. Mitochondrial fusion is controlled by the outer membrane’s mitofusin (MFN) and the inner membrane’s ocular optic atrophy 1 (OPA1). Therefore, this study sought to observe the effect of the GXN combined with IPOC on protecting against myocardial I/R injury in CRF rats and exploring the underlying mitochondrial dynamics-related mechanisms.

## Materials and Methods

### Chemicals and Reagents

Adenine containing a purity 99% was obtained from Amresco (Texas, America). The kits for determination of creatine kinase-MB (CK-MB) and cardiac troponin T (cTnT) were provided by BioSino (Beijing, China). The kits of serum creatinine (CREA) and urea nitrogen (UREA) were produced by Roche Diagnostics GmbH (Mannheim, Germany). Evans blue and triphenyltetrazolium chloride solution (TTC) were bought from Sigma-Aldrich (St. Louis, MO, United States). Tissue mitochondria isolation kit was bought from Beyotime Biotechnology Co., Ltd. (Shanghai, China). Protein concentration was measured using a bicinchoninic acid (BCA) protein assay kit (AR1189, Boster, Wuhan, China). Antibodies against FIS1 (1:500), OPA1 (1:1000), MFN1 (1:1000), and COX IV (1:1000) were purchased from Abcam (Cambridge, United Kingdom). Antibody against DRP1 (1:1000) was purchased from Cell Signaling Technology (Beverly, MA, United States). The GXN was purchased from Yabao Pharmaceutical Group Co. Ltd. (Yuncheng, China, SFDA approval number: Z14020782).

### Experimental Animals

**FIGURE 1:**
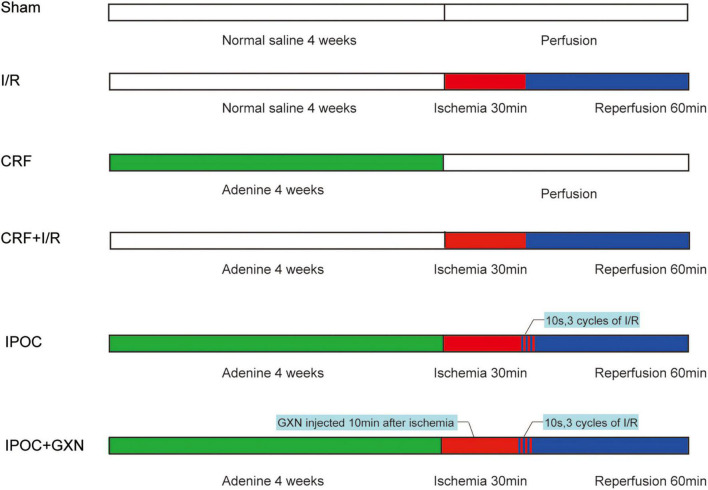
Experimental protocol. The rats were given adenine daily for 4 weeks to establish the CRF model. Then the rats underwent a 30 min ischemia procedure, followed by 60 min of reperfusion. After 30 min of ischemia, the rats were immediately given 3 cycles of reperfusion and ischemia (reperfusion: the suture line was relaxed for 10 s, ischemia: the suture line was tightened for 10 s). The GXN is injected into the iliac vein 10 min after ischemia. The sham-operated group was given an equal amount of normal saline and underwent a procedure without ischemia, but it was time-matched. Sham, sham-operated group; I/R, ischemia/reperfusion injury group (no CRF); CRF, chronic renal failure group (no I/R injury); CRF + I/R, comorbid model group; IPOC, comorbid model rat was treated with ischemic postconditioning; IPOC + GXN, the Guanxinning injection was injected in rats of the IPOC group after 10 min of ischemia.

In a survey, 90 Wistar rats (male, *n* = 45 and female, *n* = 45) aged 6 weeks weighing approximately 160–180 g were purchased by SiPeiFu Biotechnology Co., Ltd. (SCXK2011-0004, Beijing, China). Successful model rats were randomly divided into 6 groups, 15 rats each group: CRF group (CRF model was established), I/R group (myocardial I/R injury was performed), comorbid model group (CRF + I/R, myocardial I/R injury was performed in CRF rat), IPOC group (comorbid model rat was treated with IPOC), IPOC + GXN group (GXN was injected into the iliac vein in rats of the IPOC group after 10 min of ischemia). In addition, a sham operation group was set. The care and use of animals in the experiment were in accordance with the requirements of Beijing experimental animal management regulations and animal ethics regulations (Figure 1).

### Chronic Renal Failure Rat Model

The rats were given 200 mg/kg adenine daily for 4 weeks after adaptive feeding for 1 week to establish the CRF model. Rats in the sham group were given a normal diet for 4 weeks, *ad libitum* intake of normal feed, and oral gavage with an equal amount of normal saline.

### Myocardial Ischemia-Reperfusion Injury Model in Chronic Renal Failure Rat

Chronic renal failure rats were anesthetized with 1% sodium pentobarbital (50 mg/kg) by intraperitoneal injection and fixed in the supine position on the operating table. The electrocardiogram lead II was recorded. The neck and left chest skin were shaved preoperative and sterilized. The neck skin was cut open, the trachea was exposed, and the animal ventilator (tidal volume was 10 mL/kg weight, the respiratory frequency was 60 times/min) was connected to the endotracheal tube. A thoracotomy incision was made between the third and fourth rib was operated along the left edge sternum. The pericardium was cut open, and the heart was exposed. The left anterior descending coronary artery was occluded between the lower margin of the left auricle and the left margin of the pulmonary conus, and a silicone hose of diameter 1.5 mm was placed at the ligated position. Occlusion was performed for 30 min and reperfusion for 60 min. In the sham group, the left anterior descending coronary artery was not occluded.

The rats were given 200 mg/kg adenine daily for 4 weeks after adaptive feeding for 1 week to establish the CRF model. The rats in the sham operation group were given a normal diet for 4 weeks, *ad libitum* intake of normal feed, and oral gavage with an equal amount of normal saline.

### Ischemic Postconditioning Operation

After occlusion of the left anterior descending coronary artery for 30 min, the rat was immediately given 3 cycles of reperfusion and ischemia (reperfusion: the suture line was relaxed for 10 s, ischemia: the suture line was tightened for 10 s). The suture was completely released, and reperfusion was conducted for 60 min.

### Drug Intervention

**FIGURE 2:**
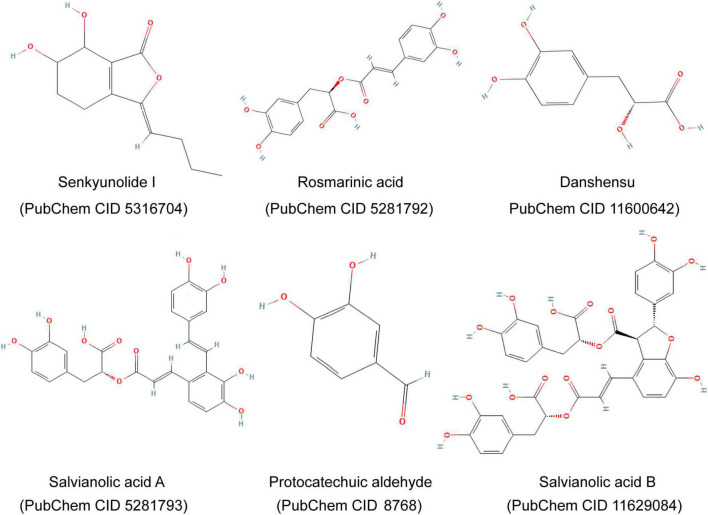
Major components and chemical structures of extracts of *Salvia miltiorrhiza Bge.* and *Ligusticum chuanxiong Hort.* (Guanxinning injection).

Guanxinning injection is composed of extracts of DS and CX and is manufactured according to Chinese Materia Medica standards, whose important ingredients include Danshensu, Salvianolic acid B, Protocatechuic aldehyde, Rosmarinic acid, Senkyunolide I, Salvianolic acid A, and so on (Figure 2; 17, 18). The GXN is injected into the iliac vein 10 min after ischemia.

### Myocardial Injury Markers and Renal Function Indexes

Two mL blood samples were collected from the abdominal aorta of rats, and serum was separated. The cTnT levels were determined by an automatic microplate reader (STAT FAX 2100, Awareness Technology, Inc., Palm City, FL, United States). CK-MB, creatinine, and urea were measured by an automatic biochemical analyzer (Hitachi 7160, Tokyo, Japan).

### Myocardial Infarction Area and Pathological Observation

The infarction area was assessed by Evans blue and TTC staining. The left anterior descending coronary artery in myocardial I/R rat models was occluded *in situ*, then 0.5% Evans blue (2–3 mL) was injected into the heart *via* the aorta. Subsequently, the heart was quickly removed and washed with physiological saline. The heart below the level of ligation was cut into five sections, which were immersed in 2% TTC phosphate buffer, incubated at 37°C for 30 min, and then fixed with 10% formaldehyde for 1 day. After the above treatment, the myocardial tissue was classified as normal myocardium (in blue), ischemic myocardium (in pale red), and infarcted myocardium (in pale white). The percentage of the area at risk (AAR, ischemic area, and infarcted area) in the left ventricular (LV) area (AAR/LV) and the percentage of the area of necrosis (AN) in the AAR (AN/AAR) were calculated by Image-Pro Plus 6.0 (Media Cybernetics, Inc., Rockville, MD, United States).

### The Morphology and Ultrastructure of the Myocardium

After fixation with 10% formalin solution, the ischemic myocardial tissue was routinely dehydrated and paraffin-embedded. Five microns-thick sections of the ischemic area were obtained (the slices are perpendicular to the long axis of the ventricle). The slices were stained with Harris alum hematoxylin staining solution and the eosin staining solution. Muscle fibers and stromal edema in ischemic myocardial tissue were observed by Olympus optical microscope (BH-2, Tokyo, Japan).

The ischemic myocardial tissue was fixed by 2.5% glutaric dialdehyde and stained by uranyl acetate and lead hydrochloride. Ultrastructural changes of mitochondria, myofibrils, and cell nuclei in cardiomyocytes were observed by Hitachi H-7650 transmission electron microscopy.

### Mitochondrial Protein Extraction and Western Blotting Detection

The ventricular part below the ligation in the rat myocardium was examined, washed repeatedly with phosphate buffer saline (PBS), and then crushed in mitochondria isolation buffer (MIB; 210 mM mannitol, 70 mM sucrose, 5 mM Tris-HCl, 1 mM EDTA, pH 7.4). Centrifugation was conducted at 600 × *g* for 10 min at 4°C, and the supernatant was retained. The process was repeated three times. Then the supernatant was centrifuged at 12,000 × *g* for 10 min at 4°C to precipitate the mitochondria. According to the weight of myocardial mitochondria, lysates (9 mM urea, 2% CHAPS, 65 mM DTT, 1 mM PMSF, 0.5% IPG buffer) of about 4 times the volume were added and suspended. The protein sample solution was quantified at a wavelength of 562 nm, following the instructions of the BCA protein assay kit.

Western blotting was used to determine changes in the expression levels of mitochondrial fission-related proteins DRP1 and FIS1 and mitochondrial fusion-related proteins OPA1 and MFN1. Briefly, after quantification, protein samples (5 μg) were separated by SDS-PAGE electrophoresis, then transferred to PVDF, sealed with 5% skim milk, and incubated with the primary antibody at 4°C overnight, followed by incubation with secondary antibodies at room temperature for 1 h. Images were then acquired using the chemiluminescent darkroom development technique. Quantification was done by densitometry of Western Blot bands using Image J software (National Institutes of Health: Bethesda, MA, United States).

### Statistical Analysis

All data were expressed as mean ± standard deviation, and SPSS software (Version 16.0, IBM Corp., Armonk, NY, United States) was used for statistical analysis. One-way analysis of variance was used to assess differences between groups. A Homogeneity of variance test was also performed, and data sets with homogeneity of variance were then submitted to the least-square deconvolution method for pairwise comparison between groups. A *P*-value < 0.05 was statistically significant.

## Results

### Myocardial Injury and Renal Function Markers

**FIGURE 3:**
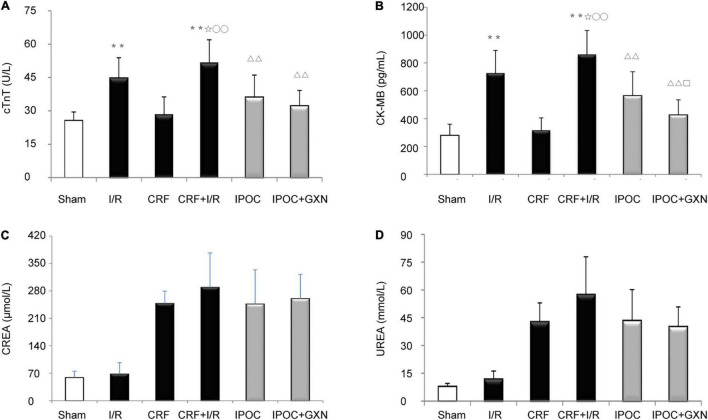
Myocardial injury markers and renal function. **(A)** cTNT; **(B)** CK-MB; **(C)** CREA; **(D)** UREA. Compared with Sham group, **P < 0.01. Compared with the I/R group, ✩*P* < 0.05. Compared with CRF group, ^○○^*P* < 0.01. Compared with CRF+IR group, ^ΔΔ^*P* < 0.01. Compared with IPOC group, ^□^*P* < 0.05.

Compared with the sham group, the myocardial injury markers, cTnT and CK-MB, were significantly increased in the I/R group rats (cTnT: 25.69 ± 3.83 U/L vs. 44.73 ± 9.16 U/L, *P* = 0.0000; CK-MB: 280.13 ± 79.98 pg/mL vs. 727.16 ± 162.68 pg/mL, *P* = 0.0000), but there was no significant difference between the sham and CRF groups (cTnT: 28.45 ± 7.87 U/L, *P* = 0.4591; CK-MB: 316.81 ± 88.15 pg/mL, *P* = 0.5900). Compared with the I/R group, the myocardial injury markers in the CRF + I/R group were significantly increased (cTnT: 51.76 ± 10.2 U/L, *P* = 0.0382; CK-MB: 860.75 ± 172.3 pg/mL, *P* = 0.0362). Compared with the CRF + I/R group, IPOC significantly reduced the expression of myocardial injury markers in the comorbid group rats (cTnT: 36.15 ± 9.90 U/L, *P* = 0.0000; CK-MB: 564.20 ± 172.34 pg/mL, *P* = 0.0000). Importantly, IPOC combined with GXN significantly reduced the expression of myocardial injury markers in the comorbid group rats (cTnT: 32.24 ± 6.96 U/L, *P* = 0.0000; CK-MB: 426.18 ± 108.21 pg/mL, *P* = 0.0000). The expression of CK-MB in the IPOC + GXN group was significantly lower than that in the IPOC group (*P* = 0.0312). Moreover, creatinine and urea levels in the IPOC and IPOC + CM groups were lower than that in the CRF + I/R group, but the difference was not statistically significant (Figure 3 and Supplementary Table 1).

### Myocardial Ischemiac and Infarcted Area

**FIGURE 4:**
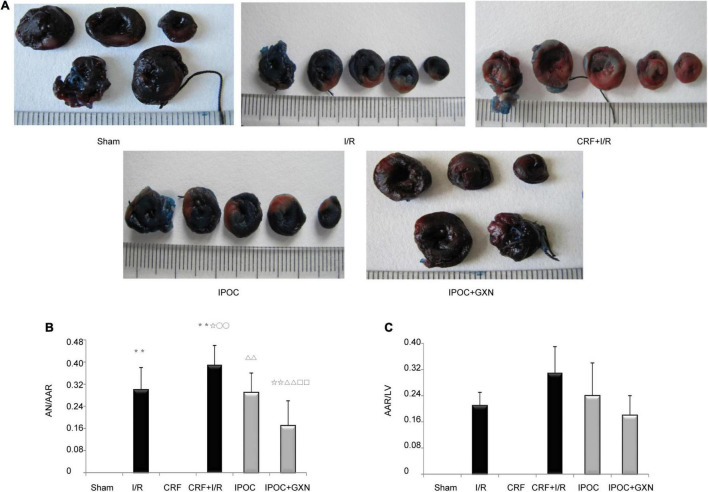
Myocardial ischemia and infarct area. **(A)** Myocardial infarction area assessed by Evans blue and Triphenyl tetrazolium chloride staining; **(B)** AN/AAR; **(C)** AAR/LV. Compared with Sham group, **P < 0.01. Compared with the I/R group, ✩*P* < 0.05, ✩✩*P* < 0.01. Compared with CRF group, ^○○^*P* < 0.01. Compared with CRF+IR group, ^ΔΔ^*P* < 0.01. Compared with IPOC group, ^□□^*P* < 0.01.

The myocardial AN/AAR in the I/R group was significantly higher than that in the sham group (0.30 ± 0.08, *P* = 0.0000). Compared with the I/R group, the AN/AAR of the myocardium in the CRF + I/R group was significantly increased (0.39 ± 0.07, *P* = 0.0175). However, compared with the CRF + I/R group, IPOC could significantly reduce the AN/AAR in the comorbidity model rats (0.29 ± 0.07, *P* = 0.0051). IPOC combined with GXN could also significantly reduce the myocardial AN/AAR of the CRF + I/R group rats (0.17 ± 0.09, *P* = 0.0000). Finally, compared with the IPOC group, the myocardial AN/AAR in the IPOC + CM group was significantly decreased (*P* = 0.0038). AAR/LV was not statistically significant between groups (Figure 4 and Supplementary Table 2).

### Morphology and Ultrastructure of the Myocardium

**FIGURE 5:**
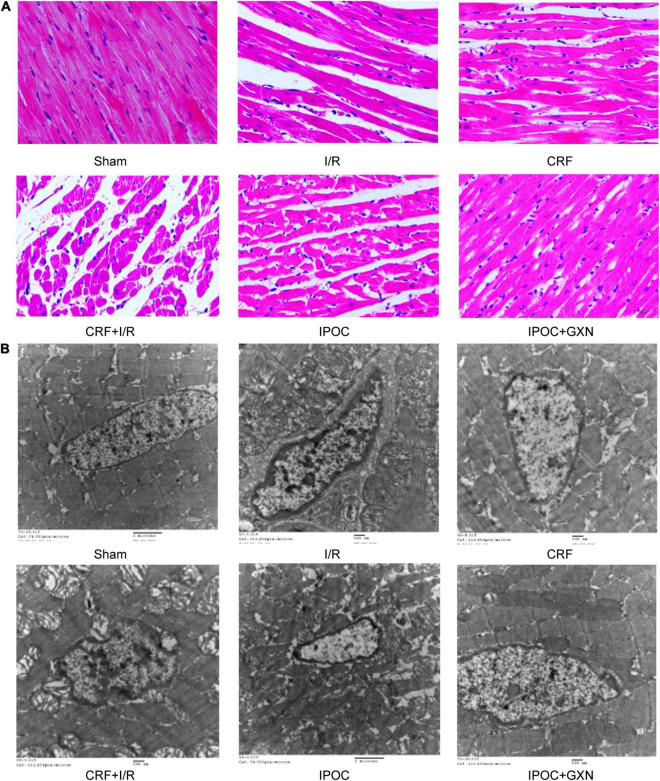
Morphology and ultrastructure of the myocardium. **(A)** HE staining; **(B)** Electron microscopical images of mitochondria.

Clear muscle fibers with regular myocardial cells and spindle-shaped nuclei were visible in the myocardial tissue of rats in the sham group with no edema or hemorrhage in the stroma. Intercellular edema was observed in myocardial tissue of the CRF group, with clear muscle fibers and myocardial cells of regular morphology. However, in the I/R group, muscle fibers were ruptured and dissolved, with significant intercellular interstitial edema, the myocardial nucleus was constricted, fragmented, or dissolved, and a large number of infiltrated inflammatory cells could be observed. Similarly, significant intercellular interstitial edema was visible in the CRF + I/R group, and the degree of muscle fiber rupture and dissolution was more obvious than in the I/R group. In the IPOC group, there were slightly swollen myocardial fibers and inflammatory cell infiltration of the stroma, which was mild compared to the CRF + I/R group. The arrangement of myocardial fibers in the IPOC + GXN group was regular, intercellular edema was milder than in the IPOC group, and the inflammatory cell infiltration was significantly alleviated (Figure 5A).

The myocardial structure in the sham and CRF groups was intact. However, in the I/R group, the myocardial structure was significantly damaged, and mitochondria exhibited lattice-shaped inclusion body formation and cristae rupture, the cellular nucleus was deformed, the nuclear membrane was contracted, the nuclear chromatin was condensed with marginal aggregation. Similarly, the myocardial structure was significantly damaged in the CRF + I/R group. However, in the IPOC group, the nucleus of the myocardium was evenly distributed, with mild edema in myocardial cells, slightly swollen mitochondria, reduced number of mitochondrial cristae, and nuclear chromatin condensation. However, no significant karyopyknosis was observed. In the IPOC + GXN group, myofibrils were arranged regularly, with no edema in the cardiomyocytes. The mitochondria exhibited an integral structure with no swelling and vesicular degeneration. Moreover, mitochondrial fusion was observed (Figure 5B).

### Mitochondrial Dynamics Protein

**FIGURE 6:**
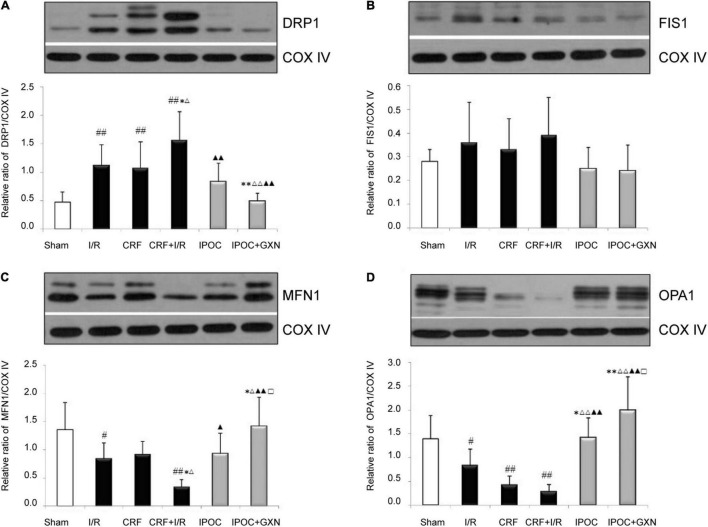
Mitochondrial dynamics protein. **(A)** DRP1; **(B)** FIS1; **(C)** MFN1; **(D)** OPA1. Compared with SHAM group, ^#^*P* < 0.05, ^##^*P* < 0.01. Compared with the I/R group, *P < 0.05, **P < 0.01. Compared with CRF group, ^Δ^*P* < 0.05, ^ΔΔ^ *P* < 0.01. Compared with CRF + I/R group, ^▲^*P* < 0.05, ^▲▲^*P* < 0.01. Compared with IPoC group, ^□^*P* < 0.05.

Compared with the sham group, the myocardial DRP1 protein levels in the I/R and CRF groups increased significantly (0.47 ± 0.18 vs. 1.11 ± 0.37, *P* = 0.0045; 0.47 ± 0.18 vs. 1.07 ± 0.46, *P* = 0.0071). Compared with the I/R groups, the myocardial DRP1 protein levels in the CRF + I/R group increased significantly (1.56 ± 0.50, *P* = 0.0388). Compared with the CRF + I/R group, both IPOC and IPOC combined with GXN could reduce the DRP1 expression in the comorbid model rats (0.83 ± 0.33, *P* = 0.0014; 0.49 ± 0.14, *P* = 0.0003). There was no significant difference in the myocardial FIS1 protein levels (Figures 6A,B).

Compared with the sham group, the myocardial MFN1 and OPA1 protein levels in the I/R group were significantly decreased (1.36 ± 0.48 vs. 0.84 ± 0.28, *P* = 0.0285; 1.39 ± 0.49 vs. 0.82 ± 0.36, *P* = 0.0452). Moreover, the myocardial MFN1 protein level in the CRF + I/R group were significantly lower than in the I/R group (0.34 ± 0.13, *P* = 0.0356), the OPA1 protein level in the CRF + I/R was also decreased, but the difference fell short of statistical significance (0.28 ± 0.15, *P* = 0.0610). IPOC could significantly increase the MFN1 and OPA1 protein levels in the comorbid model rats (0.93 ± 0.36, *P* = 0.0144; 1.41 ± 0.42, *P* = 0.0003). Compared with the CRF + I/R group, IPOC combined with GXN could also significantly increase myocardial MFN1 and OPA1 protein levels (1.42 ± 0.51, *P* = 0.0000; 1.99 ± 0.71, *P* = 0.0000). Finally, the MFN1 and OPA1 protein levels in the IPOC + GXN group were significantly higher than in the IPOC group (*P* = 0.0387, *P* = 0.0403) (Figures 6C,D).

## Discussion

Despite the growing use and success rate of coronary reperfusion techniques such as PCI, the mortality rate of AMI patients remains high, especially those with CKD (19). Myocardial I/R is widely acknowledged as a complicated process resulting from reperfusion therapy during myocardial infarction, involving metabolic and immunological variables that can potentially obliterate a significant percentage of myocardial rescue obtained by early PCI in AMI patients (20). As a result, numerous techniques to reduce reperfusion damage have been developed, including ischemic postconditioning (21). TCM has been used to treat ailments with herbal mixtures for thousands of years. Given that DS and CX can promote blood circulation and remove blood stasis, they have been used to treat blood stasis symptoms (primarily cardiovascular illnesses) in China and other Asian countries (22). GXN, which consists of extracts from the two plants mentioned above, has been established to exert remarkable cardio-renal protective effects. Danshensu is the main component of DS and reportedly alleviates cardiac I/R injury by inhibiting autophagy and apoptosis *via* activation of mammalian target of rapamycin (mTOR) signaling pathway (23). Moreover, salvianolic acid A decreases the inflammatory response in CKD and lowers platelet activation and inflammation to prevent myocardial I/R damage (24, 25). The present study found that the myocardial infarction area and myocardial injury indicators were higher in CRF rats, consistent with the literature. The IPOC intervention significantly reduced myocardial damage indicators and infarction area, and the degree of improvement was more substantial when combined with GXN, which may be attributed to enhanced mitochondrial dynamics. Although no changes in CREA and UREA were found in this study, another study revealed that GXN could reduce the elevation of blood UREA and serum cystatin C induced by transverse aortic constriction and alleviate renal pathological injury. Moreover, the metabolic mechanism of GXN in the treatment of HF with CRF by urinary metabolomics analysis showed that the cardiorenal protective mechanism of GXN was mainly related to energy metabolism and oxidative stress (26).

Mitochondrial dynamics refers to the dynamic balance between fusion and division of mitochondria. It is well-established that mitochondrial division increases, whereas fusion decreases during the I/R injury process, leading to a mitochondrial division-fusion imbalance (27). Mitochondrial fusion can reduce the stress response by combining the contents of normal mitochondria with those of partly damaged mitochondria. In contrast, mitochondrial division is required to generate new mitochondria and can be used to separate damaged mitochondria and maintain mitochondrial network quality (28). Mitochondrial fission protein DRP1 located in the cytoplasm is one of the main proteins that mediate mitochondrial division. Interestingly, DRP1 is shifted to the outside of the mitochondrial membrane during mitochondrial division and combines with the potential locus of division in mitochondria, leading to hydrolysis mitochondrial outer membrane mediated by guanosine triphosphate and promoting compression and separation of mitochondria, eventually producing two independent mitochondria (29). It is well-recognized that DRP1-dependent mitochondrial fission plays an essential role in cardiovascular disease (30). In clinically relevant large animal models of AMI, targeted inhibition of DRP1 at the onset of reperfusion was found to induce cardioprotective effects (31). It should be borne in mind that other stimuli are required to recruit DRP1 to mitochondria since it lacks a mitochondrial targeting sequence. FIS1 is one of the protein candidates implicated in the recruitment of DRP1 to the mitochondria, and its structure is similar to mitochondrial import proteins (32). Notwithstanding that FIS1 is required for mitochondrial division, studies have demonstrated that altered FIS1 expression levels do not affect DRP1 recruitment during mitochondrial division (33). Importantly, FIS1 revolves around the outer surface of the mitochondria and is not targeted at the mitochondrial future division site. Moreover, its downregulation can strongly inhibit apoptosis through multiple pathways, with a much greater degree of downregulation than DRP1 (34). Inner mitochondrial membrane (IMM) fusion and outer mitochondrial membrane (OMM) fusion represent two stages of mitochondrial fusion. It has been shown that MFN1 and MFN2 on nearby OMM mediate OMM fusion. OPA1 mediates the fusion of IMM after fusion with the outer membrane, and the mitochondrial matrix fusion generates new mitochondria (35). There is ample evidence substantiating that MFN1 and MFN2 are two MFN isoforms expressed in separate organs. MFN2 is reportedly the most abundant protein in the brain, whereas MFN1 is highly expressed in the heart (36). MFN is involved in the anchoring of apposing mitochondria prior to membrane fusion in mitochondrial fusion. In studies with differently labeled mitochondria, MFN1 tethering between molecules was shown to be more successful than MFN2 tethering in the research (37). In addition, studies have shown that OPA1 cannot promote mitochondrial fusion in MFN1-deficient cells. Similarly, MFN1 cannot promote mitochondrial elongation if OPA1 is ablated. Thus, OPA1 and MFN1 appear to be functionally dependent on each other (38, 39). Besides, substantial evidence suggests that during I/R, OPA1 and MFN protein expression are inhibited, leading to mitochondrial permeability transition pore (MPTP) opening and mitochondrial membrane permeability (MMP) phenomena, inducing apoptosis (40).

Herein, we found that the mitochondrial fission-related protein DRP1 was significantly increased in myocardial I/R injury in CRF rats. DRP1 expression was significantly decreased after treatment with IPOC. Although GXN combined with IPOC yielded a decreasing effect, the difference was not statistically significant. Meanwhile, no significant differences in FIS1 protein expression were found. Moreover, we found no significant correlation between the myocardial function of GXN combined with IPOC intervention in CRF rats after I/R injury and mitochondrial fission-related proteins DRP1 and FIS1. In addition, the myocardial mitochondrial division was significantly increased in CRF rats with I/R injury, the mitochondrial cristae were ruptured, and the expression of the mitochondrial fusion-related proteins OPA1 and MFN1 was significantly decreased. After treatment with GXN combined with IPOC, the expression of OPA1 and MFN1 proteins increased significantly, and myocardial mitochondria fusion with an integral structure was observed under a transmission electron microscope. These results provided compelling evidence that increased OPA1 and MFN1 protein expression was closely related to the protective effect induced by GXN combined with IPOC against myocardial I/R injury in uremic rats.

## Limitations

In this study, we used animals of both sexes to represent the entire population, but we did not evaluate the results for sex differences. The experimental procedures are based on relevant studies, but the short reperfusion time may affect the TTC staining results (41–43). The modeling surgery was done by one skilled researcher, but there is a high difference among the AN/AAR data, which could be related to individual variances. Indeed, this research is still preliminary and exploratory, and it does not explore the upstream signaling pathways of mitochondrial dynamics-related proteins. In the follow-up study, we will pay special attention to these issues and conduct further research.

## Conclusion

In this study, based on the observation of gross pathology, myocardial injury markers, and myocardial ultrastructure, we corroborated that *Huoxue* Chinese medicine combined with IPOC could significantly improve myocardial I/R injury in CRF rats, and the mechanism was associated to mitochondrial fusion-related proteins.

## Data Availability Statement

The original contributions presented in the study are included in the article/Supplementary Material, further inquiries can be directed to the corresponding authors.

## Ethics Statement

The animal study was reviewed and approved by Ethics Committee of Xiyuan Hospital.

## Author Contributions

AW: formal analysis, investigation, data curation, writing—original draft, and visualization. DZ and JL: conceptualization, methodology, and writing—review and editing. HYa: investigation, formal analysis, and data curation. PZ: validation, investigation, and data curation. HYu: resources. XM: supervision and project administration.

## Conflict of Interest

The authors declare that the research was conducted in the absence of any commercial or financial relationships that could be construed as a potential conflict of interest.

## Publisher’s Note

All claims expressed in this article are solely those of the authors and do not necessarily represent those of their affiliated organizations, or those of the publisher, the editors and the reviewers. Any product that may be evaluated in this article, or claim that may be made by its manufacturer, is not guaranteed or endorsed by the publisher.

## Abbreviations

AAR: area at risk
AMI: acute myocardial infarction
AN: area of necrosis
CKD: chronic kidney disease
CK-MB: creatine kinase-MB
CREA: Creatinine
CRF: chronic renal failure
cTnT: cardiac troponin T
CX: *Ligusticum chuanxiong Hort.*
DRP1: dynamin-related protein-1
DS: *Salvia miltiorrhiza Bge.*
FIS1: mitochondrial fission protein 1
GXN: Guanxinning injection
HF: heart failure
I/R: ischemia-reperfusion
IMM: inner mitochondrial membrane
IPOC: ischemic postconditioning
LV: left ventricular
MFN: mitofusin
MIB: mitochondria isolation buffer
MMP: mitochondrial membrane permeability
MPTP: mitochondrial permeability transition pore
mTOR: mammalian target of rapamycin
OMM: outer mitochondrial membrane
OPA1: ocular optic atrophy 1
PBS: phosphate buffer saline
PCI: percutaneous coronary intervention
SFDA: The State Food and Drug Administration
TTC: triphenyltetrazolium chloride solution
UREA: urea nitrogen.
