# Supplementary material for: Guanxinning Injection Combined With Ischemic Postconditioning Attenuate Myocardial Ischemic Reperfusion Injury in Chronic Renal Failure Rats by Modulating Mitochondrial Dynamics
Source: Front Cardiovasc Med. 2022 May 30;9:905254. doi: 10.3389/fcvm.2022.905254 (PMC9196273; doi:10.3389/fcvm.2022.905254)
Supplement: Supplementary file 1 [file Table_1.DOCX]

**Supplementary table1. Myocardial injury markers and renal function**

| Groups | cTnT (U/L) | CK-MB (pg/mL) | CREA (μmol/L) | UREA (mmol/L) |
| --- | --- | --- | --- | --- |
| Sham | 25.69±3.83 | 280.13±79.98 | 59.00±16.29 | 7.98±1.55 |
| I/R | 44.73±9.16^＊＊^ | 727.16±162.68^＊＊^ | 68.10±29.16 | 12.01±4.17 |
| CRF | 28.45±7.87 | 316.81±88.15 | 248.14±31.68 | 42.84±10.16 |
| CRF+I/R | 51.76±10.2^＊＊☆○○^ | 860.75±172.3^＊＊☆○○^ | 289.00±87.99 | 57.53±20.36 |
| IPOC | 36.15±9.90^△△^ | 564.20±172.34^△△^ | 246.56±87.36 | 43.46±16.72 |
| IPOC+GXN | 32.24±6.96^△△^ | 426.18±108.21^△△□^ | 260.22±62.18 | 40.22±10.61 |

Compared with Sham group, ^＊^*P*<0.05, ^＊＊^*P*<0.01. Compared with the I/R group, ^☆^*P*<0.05, ^☆☆^*P*<0.01. Compared with CRF group, ^○^*P*<0.05, ^○○^*P*<0.01. Compared with CRF+I/R group, ^△^*P*<0.05, ^△△^*P*<0.01. Compared with IPOC group, ^□^*P*<0.05, ^□□^*P*<0.01. CREA and UREA were not statistically significant between groups.
