# Supplementary material for: Guanxinning Injection Combined With Ischemic Postconditioning Attenuate Myocardial Ischemic Reperfusion Injury in Chronic Renal Failure Rats by Modulating Mitochondrial Dynamics
Source: Front Cardiovasc Med. 2022 May 30;9:905254. doi: 10.3389/fcvm.2022.905254 (PMC9196273; doi:10.3389/fcvm.2022.905254)
Supplement: Supplementary file 2 [file Table_2.DOCX]

## Table 2. Myocardial ischemia and infarct area

| Groups | AN/AAR | AAR/LV |
| --- | --- | --- |
| Sham | 0 | 0 |
| I/R | 0.30±0.08^＊＊^ | 0.21±0.04 |
| CRF | 0 | 0 |
| CRF+I/R | 0.39±0.07^＊＊☆○○^ | 0.31±0.08 |
| IPOC | 0.29±0.07^△△^ | 0.24±0.10 |
| IPOC+GXN | 0.17±0.09^☆☆△△□□^ | 0.18±0.06 |

Compared with Sham group, ^＊^*P*<0.05, ^＊＊^*P*<0.01. Compared with the I/R group, ^☆^*P*<0.05, ^☆☆^*P*<0.01. Compared with CRF group, ^○^*P*<0.05, ^○○^*P*<0.01. Compared with CRF+I/R group, ^△^*P*<0.05, ^△△^*P*<0.01. Compared with IPOC group, ^□^*P*<0.05, ^□□^*P*<0.01. AAR/LV was not statistically significant between groups.
